# ADAM8 Enhances Osteoclast Precursor Fusion and Osteoclast Formation In Vitro and In Vivo

**DOI:** 10.1002/jbmr.199

**Published:** 2010-08-03

**Authors:** Hisako Ishizuka, Verónica García-Palacios, Ganwei Lu, Mark A Subler, Heju Zhang, Christina S Boykin, Sun Jin Choi, Liena Zhao, Kenneth Patrene, Deborah L Galson, Harry C Blair, Tamer M Hadi, Jolene J Windle, Noriyoshi Kurihara, G David Roodman

**Affiliations:** 1 Department of Medicine/Hematology-Oncology and the Center for Bone Biology, University of Pittsburgh Medical CenterPittsburgh, PA, USA; 2 Department of Human and Molecular Genetics, Virginia Commonwealth UniversityRichmond, VA, USA; 3 Massey Cancer Center, Virginia Commonwealth UniversityRichmond, VA, USA; 4 National Institute of Dental and Craniofacial ResearchBethesda, MD, USA; 5 Department of Pathology, University of Pittsburgh, and Department of Medicine/Hematology-OncologyPittsburgh, PA, USA; 6 VA Pittsburgh Healthcare SystemPittsburgh, PA, USA

**Keywords:** ADAM8, TRANSGENIC/KNOCKOUT MICE, ADHESION MOLECULES, CELL DIFFERENTIATION, OSTEOCLAST

## Abstract

ADAM8 expression is increased in the interface tissue around a loosened hip prosthesis and in the pannus and synovium of patients with rheumatoid arthritis, but its potential role in these processes is unclear. ADAM8 stimulates osteoclast (OCL) formation, but the effects of overexpression or loss of expression of ADAM8 in vivo and the mechanisms responsible for the effects of ADAM8 on osteoclastogenesis are unknown. Therefore, to determine the effects of modulating ADAM expression, we generated tartrate-resistant acid phosphatase (TRAP)–*ADAM8* transgenic mice that overexpress ADAM8 in the OCL lineage and *ADAM8* knockout (*ADAM8* KO) mice. TRAP-*ADAM8* mice developed osteopenia and had increased numbers of OCL precursors that formed hypermultinucleated OCLs with an increased bone-resorbing capacity per OCL. They also had an enhanced differentiation capacity, increased TRAF6 expression, and increased NF-κB, Erk, and Akt signaling compared with wild-type (WT) littermates. This increased bone-resorbing capacity per OCL was associated with increased levels of p-Pyk2 and p-Src activation. In contrast, *ADAM8* KO mice did not display a bone phenotype in vivo, but unlike WT littermates, they did not increase RANKL production, OCL formation, or calvarial fibrosis in response to tumor necrosis factor α (TNF-α) in vivo. Since loss of ADAM8 does not inhibit basal bone remodeling but only blocks the enhanced OCL formation in response to TNF-α, these results suggest that ADAM8 may be an attractive therapeutic target for preventing bone destruction associated with inflammatory disease. © 2011 American Society for Bone and Mineral Research.

## Introduction

We previously identified *a d*isintegrin *a*nd *m*etalloprotease 8 (ADAM8) as the ADAM that was most highly expressed in osteoclast (OCL) precursors(1) and demonstrated that ADAM8 was expressed at the later stages of OCL precursor differentiation. ADAM8 significantly stimulated OCL formation in mouse marrow cultures, and knockdown of *ADAM8* levels with antisense *S*-oligonucleotides inhibited OCL formation in mouse bone marrow cultures treated with 1,25-dihydroxyvitamin D_3_ .(1) We further found that the cysteine-rich disintegrin domain of ADAM8 rather than the metalloprotease activity was responsible for its OCL stimulatory effects.(1) These results suggested that ADAM8 is an autocrine/paracrine factor that induces osteoclastogenesis.

ADAM8 also has been reported to have a role in inflammatory bone loss.(2,3) ADAM8 expression is upregulated in particle-induced arthritis,(2) and Ainola and colleagues have suggested that ADAM8 may be associated with a variety of inflammatory arthritides and mediates the bone destruction in these conditions.(3) To further clarify the role of ADAM8 in bone remodeling, we generated tartrate-resistant acid phosphatase (TRAP)–*ADAM8* (TRAP-*ADAM8* transgenic mice that overexpressed ADAM8 in cells of the OCL lineage to determine if increased expression of ADAM8 targeted to the OCL lineage results in enhanced OCL formation in vivo and *ADAM8* knockout (KO) mice to assess whether loss of ADAM8 affected normal bone remodeling. TRAP-*ADAM8* transgenic mice had increased numbers of OCL precursors that formed hypermultinucleated OCLs, decreased trabecular bone volume, and normal bone-formation levels. Enhanced expression of ADAM8 increased activation of Erk, p38 MAPK, and PI3K, which increased the bone-resorbing capacity per OCL. In addition, this increased bone resorbing capacity was associated with increased levels of p-Pyk2 and p-Src activation. In contrast, knockout of *ADAM8* in vivo did not affect normal bone remodeling but severely blunted the increase in OCL formation seen with tumor necrosis factor α (TNF-α) treatment. Taken together, these results suggest that ADAM8 is an attractive therapeutic target for blocking bone destruction in inflammatory disease.

## Materials and Methods

### Materials

Receptor activator of NF-κB ligand (RANKL) and monocyte colony-stimulating factor (M-CSF) were purchased from R&D Systems (Minneapolis, MN, USA). CD11b microbeads were obtained from Miltenyi Biotec (Gladbach, Germany). The rabbit immunoglobulin against mouse Ki67 was purchased from Dako Corp. (Carpinteria, CA, USA). All other chemicals were from Sigma Corp. (St Louis, MO, USA). Antibodies against c-Fos, NFATc1, cathepsin K, α_v_, β_3_, cFms, RANK, TRAF6, and CD44 were from Santa Cruz Biotechnology (Santa Cruz, CA, USA). Antibodies to phosphorylated and total Pyk2, Src, paxillin, Cbl, Erk1/2, JNK, p38 MAPK, Akt, and IκBα were from Cell Signaling Technology (Danvers, MA, USA), antibodies to β-actin and ATP6v0d2 were from Abcam, Inc. (Cambridge, MA, USA), and anti-DC-STAMP was from Trans Genic, Inc. (Fukuoka, Japan).

### Generation of TRAP-*ADAM8* transgenic mice

To generate mice that overexpress ADAM8 in cells of the OCL lineage, a TRAP-*ADAM8* transgene construct was generated by insertion of the 2.7-kb murine *ADAM8* cDNA (GI:19343645) into the unique *Eco*RI site of the pBSpKCR3-mTRAP vector.(4) This vector contains the murine TRAP promoter region, including 1294 bp of the 5'-flanking sequence plus the entire 5' untranslated region (UTR).(5) The vector also contains part of the second exon, the second intron, and the third exon of the rabbit *β-globin* gene, which provide both a polyadenylation site and an intron for efficient transgene expression. The 5.8-kb TRAP-*ADAM8 Xho*I fragment was microinjected into the male pronucleus of fertilized one-cell mouse embryos obtained by mating CB6F_1_ (C57BL/6 × BALB/c) males and females. Injected embryos were reimplanted into pseudopregnant CD-1 recipients, and the resulting offspring were screened by polymerase chain reaction (PCR) for the TRAP-*ADAM8* transgene using a TRAP 5'-UTR sense primer 5'-GTCCTCACCAGAGACTCTGAACTC-3' and an *ADAM8* cDNA antisense primer 5'-TCCATAAGACGCTGAGCAGCCAG-3'. Southern blot analysis of transgenic founders was performed to verify transgene structural integrity and to determine transgene copy number. TRAP-*ADAM8* transgenic mouse lines were maintained on a CB6F_1_ background. Similar results were obtained in two independent TRAP-*ADAM8* transgenic lines.

### Generation of *ADAM8* KO mice

To generate *ADAM8* KO (*ADAM8*^*−/−*^) mice, an *ADAM8* targeting vector was derived from pKO NTKV-1901, which contains both a PGK/*neo*/BGH cassette for positive selection of homologous recombinants with G418 and an MC1*-tk* cassette for negative selection of nonhomologous recombinants with ganciclovir (Stratagene, La Jolla, CA, USA). *LoxP* sites were inserted at both ends of the PGK/*neo*/BGH cassette, and 129/SV genomic fragments containing *ADAM8* exons 1 to 17 (nucleotides –1543 to 7736 of GI:2326260) and 18 to 22 (7737 to 10713) were inserted at the 5' and 3' ends of the *neo* cassette, respectively. A third *loxP* site was introduced into the *Eco*RI site (3599) in intron 5. The *ADAM8* targeting vector was linearized with Ssp I and electroporated into 129/Sv embryonic stem (ES) cells. Genomic DNA from ES cell clones resistant to both G418 and ganciclovir was screened for homologous recombination by long-range PCR with BGH sense and *ADAM8* intron 22 antisense (10782 to 10759) primers. Southern blot analysis was performed on clones that were positive by PCR and retained the intron 5 *loxP* site using a PGK/*neo* probe to verify clone purity and integrity of both genomic arms. C57BL/6 blastocysts were injected with an ES cell clone carrying a correctly targeted *ADAM8* allele and implanted into pseudopregnant CD-1 recipients. The resulting male chimeras were bred to C57BL/6 females, and tail genomic DNA from agouti offspring was screened by PCR for the presence of the targeted *ADAM8* allele.

Mice carrying the targeted allele were bred to FVB/N-Tg (EIIa-*cre*) mice that express Cre in the germ line (Jackson Laboratory, Bar Harbor, ME, USA). Cre-mediated recombination generated both *ADAM8*^*+/*−^ mice carrying a standard KO allele in which exons 6 to 17 have been deleted and *ADAM8*^*+/*−^ mice possessing a conditional knockout allele containing *loxP* sites in introns 5 and 17. The conditional KO mice were not used in this study. *ADAM8*^*+/*−^ mice were interbred to generate mice of the *ADAM8* genotypes (+/ +, +/−, and −/−) used in this study. Offspring were screened by PCR using a common exon 4 primer (3297−3320: 5'-GGCTCAAGCTACACAGAGACCTAC-3'), a WT-specific intron 5 primer (3709−3686: 5'-GGCTACACCGAAAAACCCTGTCTC-3'), and a KO-specific exon 18 primer (7996−7974: 5'-GACAGTGGCACTCCCTCTTGTGG-3'). A Southern blot of *ApaLI/PflFI* double restriction digests of genomic DNA from offspring of each of the genotypes was probed with an *ADAM8* genomic fragment (−1543 to −277) to verify deletion of exons 6 to 17 in the KO mice (data not shown). The *ADAM8*^−/−^ mouse line was maintained on a C57BL/6 × 129/Sv mixed background.

All animal studies were approved by the Institutional Animal Care and Use Committees at the University of Pittsburgh School of Medicine, the VA Pittsburgh Healthcare System, and Virginia Commonwealth University and were conducted in accordance with the Animal Welfare Act, the PHS Policy on Humane Care and Use of Laboratory Animals, and the *U.S. Government Principles for the Utilization and Care of Vertebrate Animals Used in Testing, Research, and Training*.

### Quantitative µCT measurements

The gross morphologic and microarchitectural traits of the distal area of the femur and L_5_ vertebra were examined by quantitative µCT. The L_5_ vertebrae were used to assess histomorphometry of the trabecular bones, and the femurs were used to measure mean cortical thickness. Specimens were held with Styrofoam within plastic vials and positioned within a 25-mm-diameter acrylic tube. After an initial scout scan, full-length scans were obtained at an isotropic voxel resolution of 10.5 µm using a commercial scanner (Scanco Viva CT40, Scanco Medical AG, Bassersdorf, Switzerland) using the following settings: Energy = 55 kVp, current = 145 mA, and integration time = 300 ms. A total of 300 slices with an increment of 25 µm were obtained on each bone sample starting 1.0 mm below the growth plate in the area of the secondary spongiosa. The area for analysis was outlined within the trabecular compartment, excluding the cortical and subcortical bone. Every 25 sections were outlined, and the intermediate sections were interpolated with the contouring algorithm to create a volume of interest. Segmentation values used for analysis were sigma 0.8, support 1, and threshold 275. A 3D analysis was done to determine bone volume (BV/TV, %), trabecular number (Tb.N, /mm^2^), trabecular thickness (Tb.Th, mm), and trabecular bone spacing (Tb.Sp, mm). Cortical bone also was measured on the femur 2 mm below the growth plate, and the same segmentation parameters were used for analysis.

### Bone histomorphometric analyses

Mouse spines and femurs were fixed overnight in 10% buffered formalin, decalcified in 10% EDTA, embedded in paraffin, and then sectioned and stained for TRAP activity or with Masson's trichrome. Bone histomorphometric parameters from femurs were determined by measuring the areas at least 0.5 mm from the growth plate, excluding the primary spongiosa and trabeculae connected to the cortical bone. OCL number per bone surface (NOc/BS, N/mm^2^) and the percentage of OCL surface to bone surface (OcS/BS, %) were obtained by counting TRAP^+^ cells and by measuring the area in serial 5-µm-thick sagittal histologic sections of femurs from 20-week-old WT mice. TRAP^+^ cells forming resorption lacunae on the surface of the trabeculae and containing one or more nuclei were identified as OCLs. Mineral apposition rates and bone-formation rates were measured in trabecular bone of the vertebral bodies. A real measurement and a surface measurement were made by grid-intersect counting on calibrated digital images by observers blinded to the treatment group being examined. The abbreviations for histomorphometric parameters were according to the recommendation by the American Society of Bone and Mineral Research Histomorphometry Nomenclature Committee.(6)

### In vitro osteoclastogenesis in cultures of purified early OCL precursors

Whole bone marrow cells were flushed from long bones of 4- to 20-week-old WT, TRAP-*ADAM8* or *ADAM8*^−*/*−^ mice and plated on 100-mm tissue culture plates in α-Minimal Essential Medium (α-MEM, Gibco BRL Invitrogen, Carlsbad, CA, USA) containing 10% fetal bovine serum (FBS, Invitrogen). Cells were incubated at 37°C in 5% CO_2_ overnight. Nonadherent cells were harvested, and then CD11b^+^ mononuclear cells obtained using Miltenyi Biotec MACS (Magnetic Cell Sorting) system.(7) CD11b^+^ cells then were cultured in α-MEM containing 10% FBS plus 10 ng/mL of monocyte colony-stimulating factor (M-CSF) for 2 to 3 days. These CD11b^+^ cells were used as a source of enriched early OCL precursors. CD11b^+^ cells then were cultured in α-MEM containing 10% FBS in the presence of 50 ng/mL of RANKL (R&D Systems) for 3 to 4 days to generate OCLs, and the level of OCL formation was determined by counting the number of TRAP^+^ multinucleated cells (≥3 nuclei/cell).Immunoblotting of extracts from OCL precursors obtained from TRAP-*ADAM8*, *ADAM8*^−/−^, or WT mice

Lysates from RANKL-treated or control OCL precursors from TRAP-*ADAM8*, *ADAM8*^−*/*−^, or WT mice were processed for Western blot analysis as described previously.(8) Membranes then were exposed to primary antibodies overnight at 4°C, washed three times, and incubated with secondary goat antimouse or antirabbit IgG horseradish peroxidase (HRP)–conjugated antibody for 1 hour. Membranes were washed extensively, and an enhanced chemiluminescence detection assay was performed following the manufacturer's directions (Pierce Biotechnology, Rockford, IL, USA). All blots were quantitated densitometrically, and the results were expressed relative to control and normalized to β*-*actin.

### In vitro bone resorption assays of cultured OCLs

CD11b^+^ cells were cultured on mammoth dentin slices (Wako, Osaka, Japan) in α-MEM + 10% FCS containing M-CSF and RANKL. After 14 days of culture, the cells were removed, the dentin slices were stained with acid hematoxylin, and the areas of dentin resorption were determined using image-analysis techniques (NIH Image System).

### Treatment of *ADAM8*^−*/*−^ mice with TNF-α

Three-month-old *ADAM8*^−/−^ (*n* = 8) and WT mice (*n* = 7) were injected over the calvaria for 5 consecutive days with TNF-α (1.5 µg in 50 µL of PBS/day) or PBS alone. On day 6, calvaria were harvested, fixed in 10% buffered formalin, and processed for histology, and tissue sections stained for TRAP activity and analyzed as described earlier.

### Statistical analysis

For all cell culture studies, significance was evaluated using a two-tailed unpaired Student's *t* test, with *p* < .05 considered to be significant. For the histologic analysis of bones, two-way analysis of variance (ANOVA) was performed to compare the differences in all histomorphometric variables among genotypes and across age. One-way ANOVA was performed to compare the differences among age groups of each genotype of variables in which an age-related change was detected. Statistical analysis was performed using NCSS 2004 and PASS programs (NCSS, Kaysville, UT, USA).

## Results

### TRAP*-ADAM8* transgenic mice have increased OCL formation and decreased trabecular bone volume

**Fig. 1:**
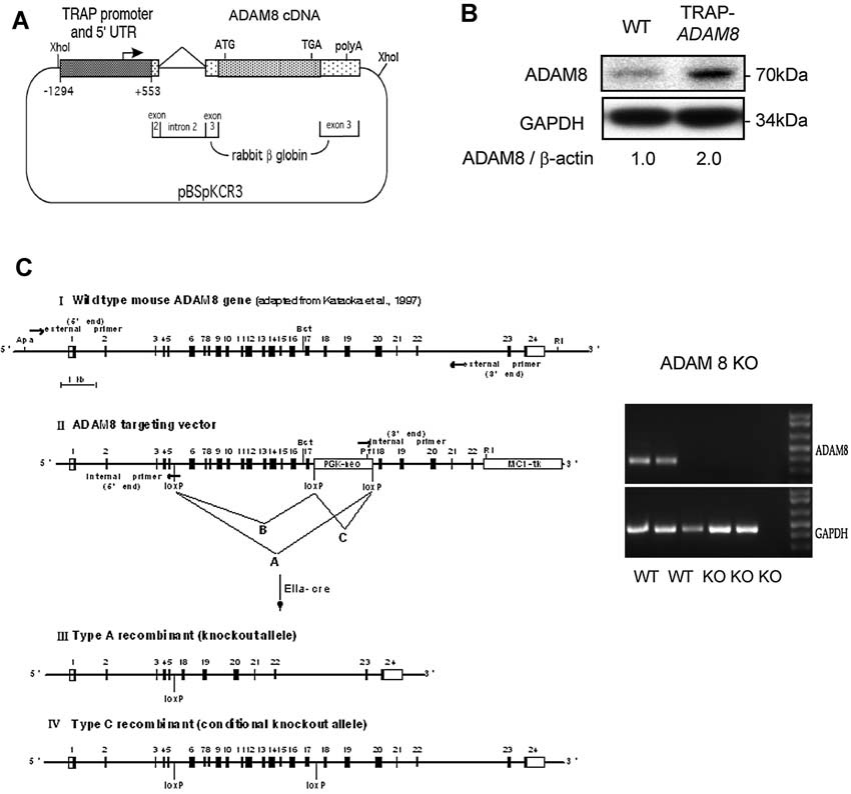
Generation of TRAP-*ADM8* transgenic mice. (*A*) Map of the TRAP-*ADAM8* transgene construct. (*B*) *ADAM8* expression levels in OCL precursors from a WT mouse and offspring from TRAP-*ADAM8* mice. The bone marrow cells were cultured with M-CSF for 3 days, and then cells were collected in commercial lysate buffer (Santa Cruz Biotechnology). ADAM8 expression was determined by Western blot analysis using specific anti-ADAM8 antibody (Millipore Corporation, Temecula, CA, USA). Similar results were detected in three different lines of mice. (*C*) Targeting vector for generation of both conditional and total knockout of the *ADAM8* gene. Structure of the (*I*) WT targeted *ADAM8* allele and (*II*) the targeting vector. The positions of the bovine growth hormone (BGH) and intron 22 primers used for initial screening of ES cell clones are shown. (*III*) Structure of the knockout or (*IV*) conditional knockout allele of *ADAM8* following breeding of mice carrying the initially targeted allele to EIIa-*cre* transgenic mice. The lack of *ADAM8* mRNA expression was verified in three knockout lines of mice by semiquantitative RT-PCR. *GAPDH* mRNA also was amplified from each sample as a control for RNA quantity and integrity.

We assessed the effects of ADAM8 overexpression on OCLs in vivo by targeting expression of the murine *ADAM8* cDNA to cells in the OCL lineage using the murine TRAP promoter (Fig. 1*A*), which directs efficient expression to OCLs and OCL precursors.(5) OCL precursors from these mice then were screened for *ADAM8* expression by Western blot analysis using a specific antibody to ADAM8. OCL precursors from TRAP-*ADAM8* mice showed approximately 2-fold greater *ADAM8* expression than WT mice (Fig. 1*B*). In addition, we generated an *ADAM8*^−*/*−^ mouse as depicted in Fig. 1*C* to examine the consequence of *ADAM8* deficiency on bone in vivo.

**Fig. 2:**
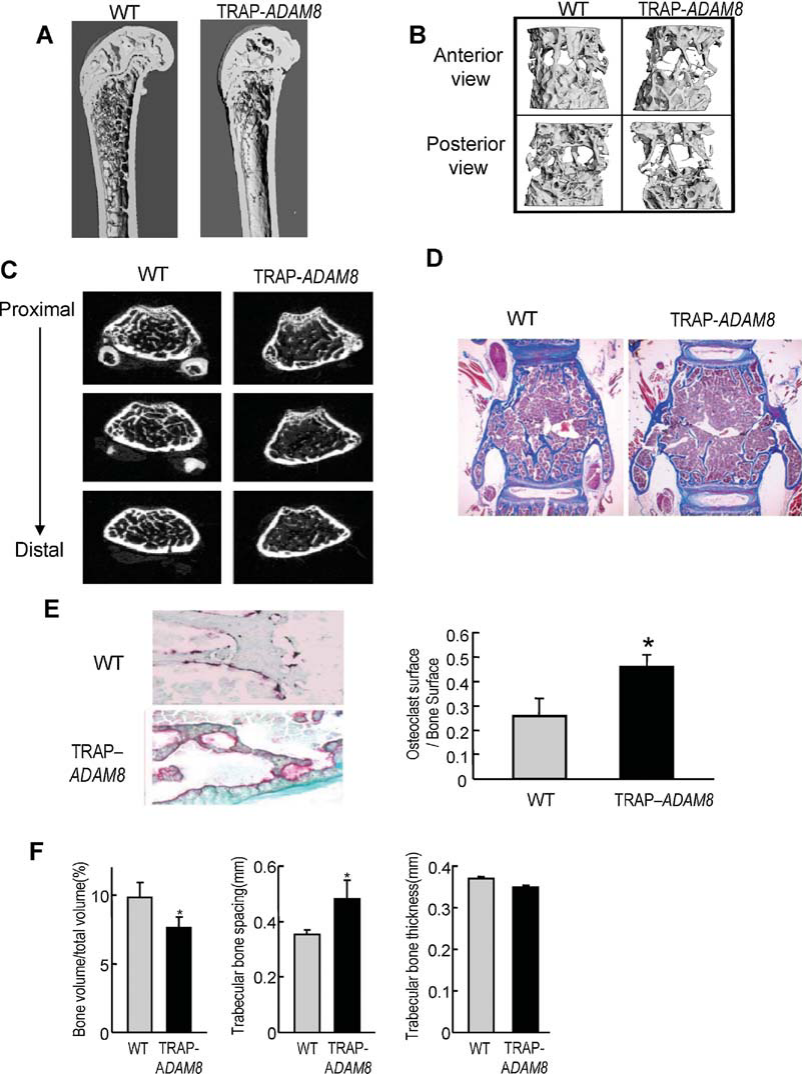
Characterization of bones from *ADAM8* transgenic mice. (*A*) Quantitative µCT analysis of trabecular areas of the femurs at 30-week-old mice. (*B*) Quantitative µCT analysis of the fifth lumbar vertebrae at 30-week-old mice (anteroposterior view). (*C*) Quantitative µCT analysis of the fifth lumbar vertebrae for 30-week-old mice (proximal-distal view). (*D*) Histologic features of a TRAP-*ADAM8* mouse compared with a WT control mouse at 20 weeks of age. TRAP staining, counterstained with methyl green–thionin. Original magnification ×100. (*E*) Osteoblast surface/bone surface *n* = 5 mice per group. *Significant differences (*p* < .05) between TRAP-*ADAM8* and WT mice by Mann-Whitney test. Data represent results from a typical experiment. Similar results were seen in three independent experiments. *p < .05 compared with WT mice. (*F*) Quantitation of µCT analysis of the fifth lumbar vertebrae from 30-week-old WT and TRAP-*ADAM8* mice. The percent bone volume per total volume, trabecular bone spacing (mm), and trabecular bone thickness (2 mm) are shown. Data represent mean values ± SEM for 15 mice per group, significantly different from WT mice by Mann-Whitney test, *p* < .05.

Quantitative µCT analysis of trabecular areas of the femurs (Fig. 2*A*) and 5th lumbar vertebrae (Fig. 2*B, C*) from TRAP-*ADAM8* and WT mice revealed the presence of osteopenia in the TRAP-*ADAM8* mice. As shown in Fig. 2*C, D*, the vertebral bodies from TRAP-*ADAM8* mice showed markedly decreased total bone volume compared with WT mice. Morphometric analysis confirmed that the OCL surface was significantly increased in TRAP-*ADAM8* mice compared with WT mice (Fig. 2*E*). Bone volume/total bone volume, trabecular number, and trabecular thickness were decreased significantly in TRAP-*ADAM8* mice, whereas trabecular spacing was increased (Fig. 2*F*). However, there was no significant difference in bone formation or mineral apposition rates (data not shown) between TRAP-*ADAM8* and WT mice. µCT analysis of the fifth vertebrae from TRAP-*ADAM8* mice showed that the trabecular bone fraction [bone area/total area (BAr/TAr)] was 40% ± 7% versus 59% ± 3.5% for WT mice.

### OCL precursors from TRAP-*ADAM8* mice form increased numbers of OCLs in response to RANKL

**Fig. 3:**
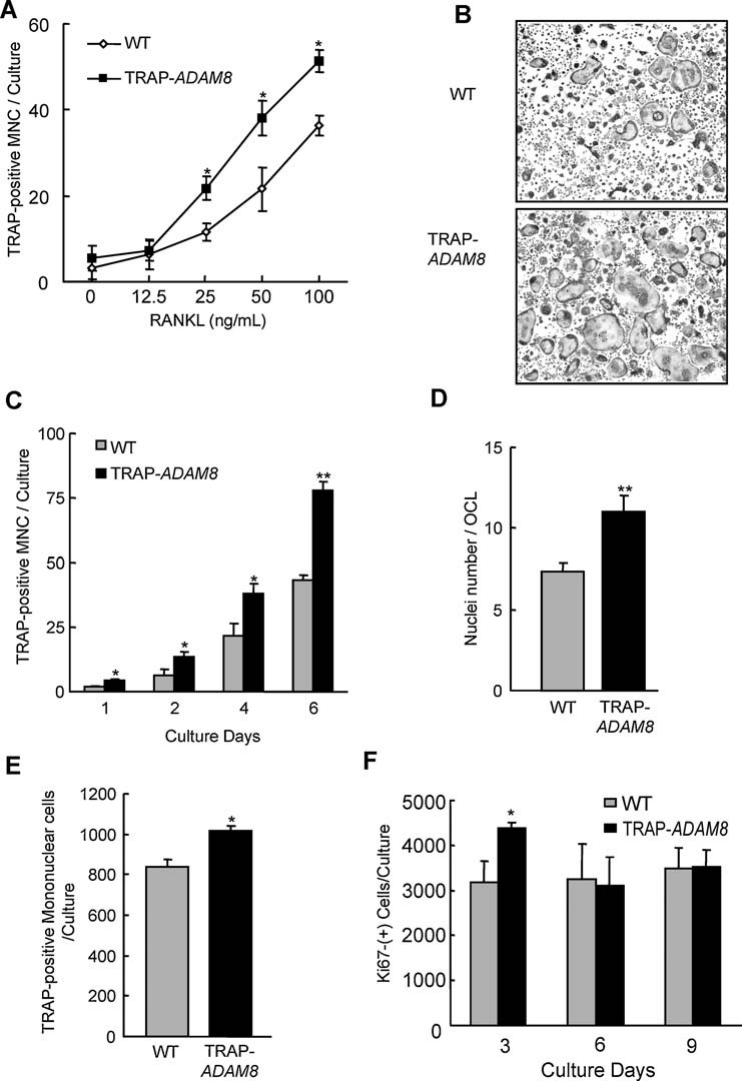
OCL precursors from TRAP-*ADAM8* mice for increased numbers of OCL in response to RANKL compared with WT mice. (*A*) Number of TRAP^+^ multinuclear cells per well. Highly purified CD11b^+^ cells from WT and TRAP-*ADAM8* mice were cultured as described in “Materials and Methods.” Results are shown as the mean ± SD. *p < .01, significantly different from WT. Similar results were seen in four independent experiments. (*B*) TRAP staining of OCLs formed by nonadherent marrow cells. Marrow cells (2 × 10^5^/well) from WT and TRAP-*ADAM8* mice were cultured with M-CSF (10 ng/mL) and RANKL (50 ng/mL) for 7 days. Note the increased number and size of the OCLs formed in the TRAP-*ADAM8* cultures. Original magnification ×200. (*C*) Time course for OCL formation by TRAP-*ADAM8* and WT OCL precursors. CD11b^+^ cells from WT and TRAP-*ADAM8* mice were cultured in the presence of M-CSF (10 ng/mL) and RANKL (50 ng/mL). After 1, 2, 4, or 6 days, after addition of RANKL, cells were stained with TRAP, and TRAP^+^ multinucleated cells were counted. Results are shown as the mean ± SD for quadruplicate culture. *p < .05 and **p < .01, significantly different from WT. Similar results were seen in three independent experiments. (*D*) Nuclear number per OCL for TRAP-*ADAM8* and WT OCLs. Nuclei per TRAP^+^ OCLs were scored in 100 OCLs counted at random. Results are shown as the mean ± SD. *p < .01, significantly different from WT. Similar results were seen in three independent experiments. (*E*) Number of TRAP^+^ mononuclear cells are increased in TRAP-*ADAM8* cultures. CD11b^+^ cells from WT and TRAP-*ADAM8* mice were cultured in the presence of M-CSF (10 ng/mL) and RANKL (50 ng/mL). After 4 days of culture, cells were stained with TRAP, and TRAP^+^ mononuclear cells were counted. Results are shown as the mean ± SD. *p < .01, significantly different from WT. Similar results were seen in three independent experiments. (*F*) Ki67^+^ mononuclear cells are increased in cultures from TRAP-*ADAM8* mice. CD11b^+^ cells from WT and TRAP-*ADAM8* mice were cultured in the presence of M-CSF (10 ng/mL) and RANKL (50 ng/mL). After 3, 6, or 9 days of culture, cells were evaluated by immunostaining using an anti-Ki67 antibody. All results are shown as the mean ± SEM for quadruplicate culture. *p < .05, significantly different from WT. Similar results were seen in two independent experiments.

CD11b^+^ marrow cells from TRAP-*ADAM8* mice formed increased numbers of OCLs, which contained increased nuclei/OCL compared with WT littermates (Fig. 3*A–D*). OCL formation in marrow cultures from TRAP-*ADAM8* mice was evident as early as 1 day after RANKL addition and remained elevated through day 6 compared with WT cultures (Fig. 3*C*). Further, OCL precursor numbers and proliferation, as measured by TRAP^+^ mononuclear cells and Ki67 staining, respectively, was increased significantly in marrow cultures from TRAP-*ADAM8* mice compared with WT cultures during the early phases (days 3 to 4) of culture (Fig. 3*E, F*). However, subsequently, the levels of Ki67 positivity of OCL precursors on days 6 and 9 were similar in TRAP-*ADAM8* and WT cultures (Fig. 3*F*). Similarly, [^3^H]thymidine incorporation in OCL precursors was increased modestly in marrow cultures from TRAP-*ADAM8* mice compared with WT mice (1949 ± 135 versus 1554 ± 78 cpm/well, *p* < .05).

### Expression of OCL differentiation markers is enhanced in TRAP-*ADAM8* OCL precursors

**Fig. 4:**
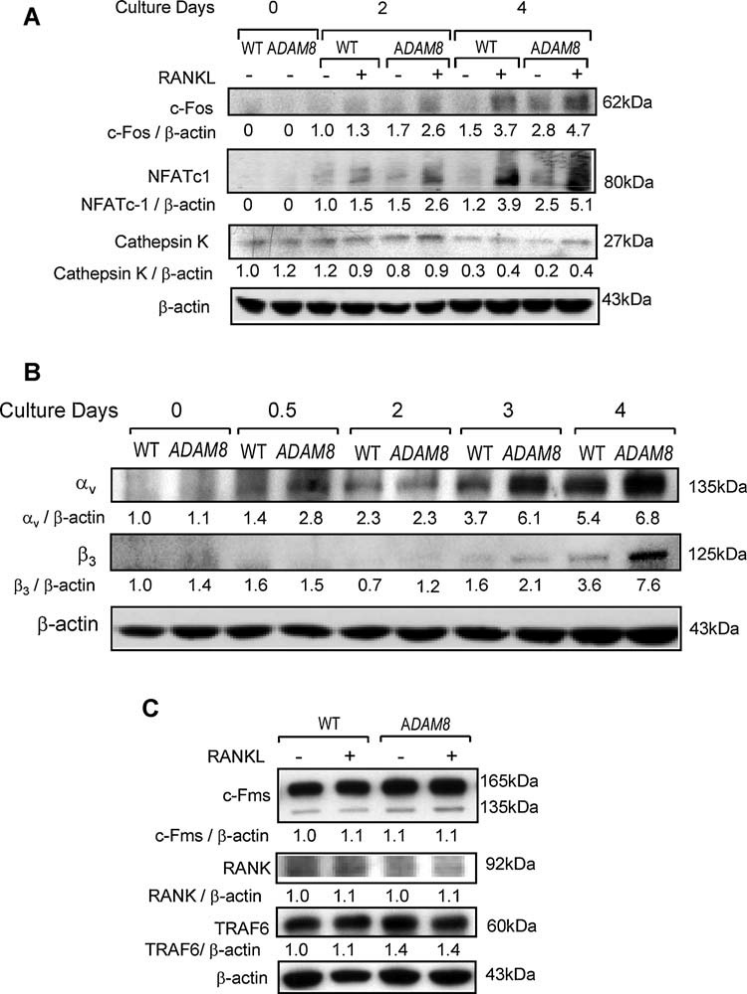
Expression of OCL differentiation markers by TRAP-*ADAM8* OCL precursors treated with RANK. (*A*) c-Fos, NFATc-1, and cathepsin K expression. CD11b^+^ OCL precursors from WT and TRAP-*ADAM8* mice were cultured with M-CSF for 3 days and then with 50 ng/mL of RANKL for the indicated times, and whole-cell lysates (30 µg of protein/lane) were subjected to immunoblot analysis using anti-c-Fos, anti-NFATc-1, or anti–cathepsin K antibodies. β-Actin is shown as a loading control. Similar results were seen in two independent experiments. (*B*) α_v_ β_3_ expression by OCL precursors from WT and TRAP-*ADAM8* mice. OCL precursors were cultured with RANKL (50 ng/mL) for the indicated times, and whole-cell lysates (30 µg of protein/lane) were subjected to immunoblot analysis using anti-α_v_ or anti-β_3_ antibody. β-Actin is shown as a loading control. Similar results were seen in two independent experiments. (*C*) c-Fms, RANK, and TRAF6 expression by OCL precursors from TRAP-*ADAM8* mice. OCL precursors from WT and TRAP-*ADAM8* mice were cultured with RANKL (50 ng/mL) for 3 days, and whole-cell lysates (30 µg of protein/lane) were subjected to immunoblot analysis using anti-c-Fms, anti-RANK, or anti-TRAF6 antibody. TRAF6 but not RANK or c-Fms was increased in OCL precursors from TRAP-ADAM8 mice. β-Actin is shown as a loading control. Similar results were seen in three independent experiments.

As shown in Fig. 4*A*, marrow cultures from TRAP-*ADAM8* mice expressed relatively higher levels of c-Fos and NFATc-1 compared with WT mice on days 2 and 4 after addition of RANKL. α_v_ β_3_ integrin expression also was increased in TRAP-*ADAM8* OCLs compared with WT OCLs starting at day 3 of culture (Fig. 4*B*). The levels of TRAF6 but not expression of c-Fms and RANK were increased 1.4 ± 0.1 (mean ± SEM)–fold in OCL precursors from TRAP-*ADAM8* mice compared with WT mice (Fig. 4*C*).

### Src tyrosine kinase is activated in TRAP-*ADAM8* OCL precursors

**Fig. 5:**
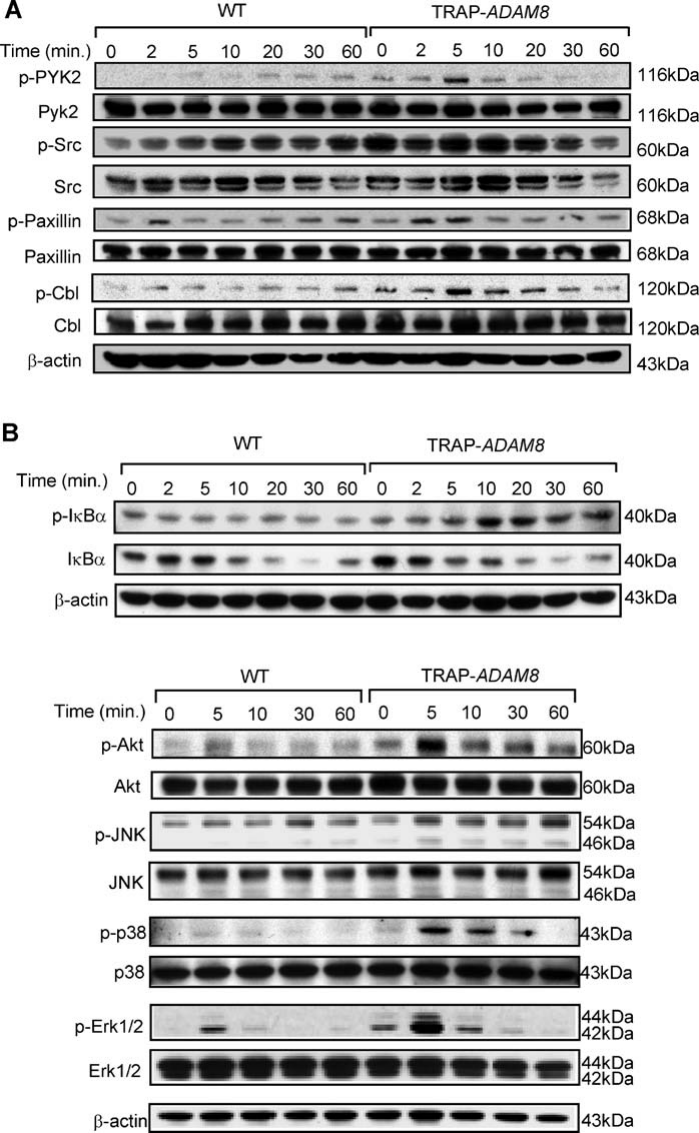
Activation of signaling pathways involved in OCL formation and activity in TRAP-*ADAM8* and WT OCL precursors. (*A*) Activation of tyrosine kinases in OCL precursors. OCL precursors from WT and TRAP-*ADAM8* mice were cultured with M-CSF for 3 days and then 50 ng/mL of RANKL for the indicated times, and whole-cell lysates (30 µg of protein/lane) were subjected to immunoblot analysis using antibodies recognizing phosphorylated and total signaling molecules of Pyk2 (Tyr402), Src (Tyr416), paxillin (Tyr118), and Cbl (Tyr731). β-Actin is shown as a loading control. (*B*) Activation of the NF-κB, Akt, and MAPK signaling pathways in OCL precursors from TRAP-*ADAM8* mice. OCL precursors from WT and TRAP-*ADAM8* mice were cultured with 50 ng/mL of RANKL for the indicated times, and whole-cell lysates (30 µg of protein/lane) were subjected to immunoblot analysis using antibodies recognizing phosphorylated and total IκBα (Ser32), Akt (Ser473), JNK (Thr183/Tyr185), p38 MAPK (Thr180/Tyr182), and Erk1/2 (Thr202/try204). β-Actin is shown as a loading control.

Since ADAM8 binds its cognate receptor, α_9_ β_1_ integrin, on OCL precursors,(9) we then measured integrin signaling in OCL precursors from TRAP-*ADAM8* mice by determining if Pyk2 (Tyr402), Src (Tyr416), Cbl (Tyr731), and paxillin (Tyr118) were activated in whole-cell lysates of OCL precursors from TRAP-*ADAM8* mice by Western blot analysis. Increased phosphorylation of Pyk2 and Cbl in TRAP-*ADAM8* cells was detected at 5 minutes and then slowly decreased, whereas p-Pyk2 and p-Cbl appeared slowly over 60 minutes in WT cells (Fig. 5*A*). Src activation also was increased in TRAP-*ADAM8* OCL precursors compared with WT OCL precursors (Fig. 5*A*). The increased phosphorylation of Src was detected at the initiation of the cultures and lost by 60 minute in TRAP-*ADAM8* OCL precursors after incubation with RANKL, whereas there was no change in total Src. Paxillin phosphorylation was biphasic in WT cells with a peak at 2 minutes, after which it disappeared rapidly and reemerged at 20 minutes. In the TRAP-*ADAM8* OCL precursors, the initial p-paxillin peak was stronger and longer, whereas Pyk2, Cbl, and paxillin levels were similar in OCLs from TRAP-*ADAM8* and WT mice (Fig. 5*A*).

### Activation of the Akt, MAPKs, and NF-κB signaling pathways is increased in TRAP-*ADAM8* OCL precursors

We then determined which of the multiple signaling pathways implicated in the induction of OCL formation(10,11) was activated by overexpression of ADAM8 in OCL precursors. As shown Fig. 5*B*, there was increased NF-κB activation in TRAP-*ADAM8* cells compared with WT cells with RANKL, as noted by increased phosphorylation and faster degradation of IκBα in TRAP-*ADAM8* OCL precursors. The phosphorylation levels of the MAPKs, Erk1/2, and p38 MAPK, and AKT (an indicator of PI3K activation) also were greater and were more rapid when OCL precursors from TRAP-*ADAM8* mice were treated with RANKL for the indicated times compared with WT mice (Fig. 5*B*). RANKL induced Erk1/2 and Akt activation in both WT and TRAP-*ADAM8* OCL precursors, exhibited a sharp peak at 5 minutes, disappearing rapidly, but the degree of activation was significantly higher in TRAP-*ADAM8* cells. The pattern of activation of p38 MAPK was similar to Erk1/2 and Akt except that the loss of p-p38 was slower. In contrast, JNK1 phosphorylation/activation induced by RANKL was similar in TRAP-*ADAM8* and WT OCL precursors.

### DC-STAMP is increased on TRAP-*ADAM8* OCL precursors

**Fig. 6:**
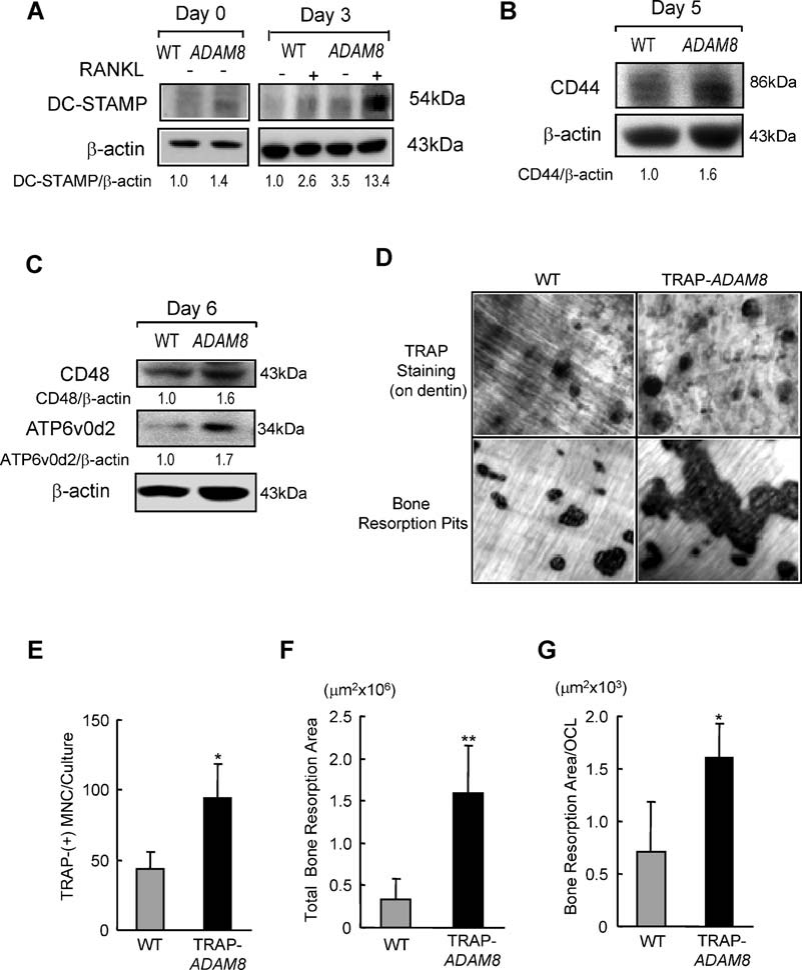
Expression of fusion molecules by OCL precursors and bone resorption by OCLs from TRAP-*ADAM8* and WT mice. OCL precursors from WT and TRAP-*ADAM8* mice were cultured with M-CSF for 3 days and then 50 ng/mL of RANKL for the indicated times, and whole-cell lysates (30 µg of protein/lane) were subjected to immunoblot analysis using (*A*) anti–DC-STAMP, (*B*) anti-CD44, and (*C*) anti-CD48 or anti-ATP6v0d2 antibodies. β-Actin is shown as a loading control. Similar results were seen in two independent experiments. (*D*) Bone-resorption lacunae formed by WT and TRAP-*ADAM8* OCLs. OCL precursors were cultured on dentin slices with 30 ng/mL of M-CSF and 100 ng/mL of RANKL for 14 days. At the end of the culture period, the dentin slices were stained with acid hematoxilin to display resorption pits. Original magnification ×200. (*E*) The number of TRAP^+^ OCLs per dentin slice. (*F*) Resorption area per dentin slice. (*G*) Bone-resorption area per OCL. All results are shown as the mean ± SD for at least triplicate cultures. *p < .05, significantly different from WT N.S. = not significantly different compared with WT. Similar results were seen in three independent experiments.

Since OCL precursors from TRAP-*ADAM8* mice formed OCLs with increased nuclei per OCL, we determined the expression levels of several fusion molecules known to be expressed in OCL precursors. OCL precursors from TRAP-*ADAM8* mice treated with RANKL had increased expression of DC-STAMP (Fig. 6*A*). After 3 days of RANKL treatment, when DC-STAMP expression peaked in OCL precursor cultures, TRAP-*ADAM8* OCL precursors expressed threefold higher levels of DC-STAMP than medium-treated TRAP-*ADAM8* OCL precursors (Fig. 6*A*) and fivefold higher than RANKL-treated WT OCL precursors (Fig. 6*A*). The expression levels of CD44, CD48, and ATP6v0d2, which peaked on days 5 and 6 of OCL precursor cultures, were only modestly elevated in TRAP-*ADAM8* OCL precursors compared with WT OCL precursors (Fig. 6*B, C*).

### Bone resorption by TRAP-*ADAM8* OCLs is increased compared with WT OCLs

Bone resorption in marrow cultures from TRAP-*ADAM8* mice was increased fivefold compared with WT cultures (Fig. 6*D, F*). The increased resorption in TRAP-*ADAM8* cultures was not due solely to there being more OCLs formed than in WT cultures (Fig. 6*E*) but also was due to increased bone resorption per OCL (twofold) compared with WT cultures (Fig. 6*G*).

### *ADAM8* KO mice do not increase OCL formation in response to TNF-α

**Fig. 7:**
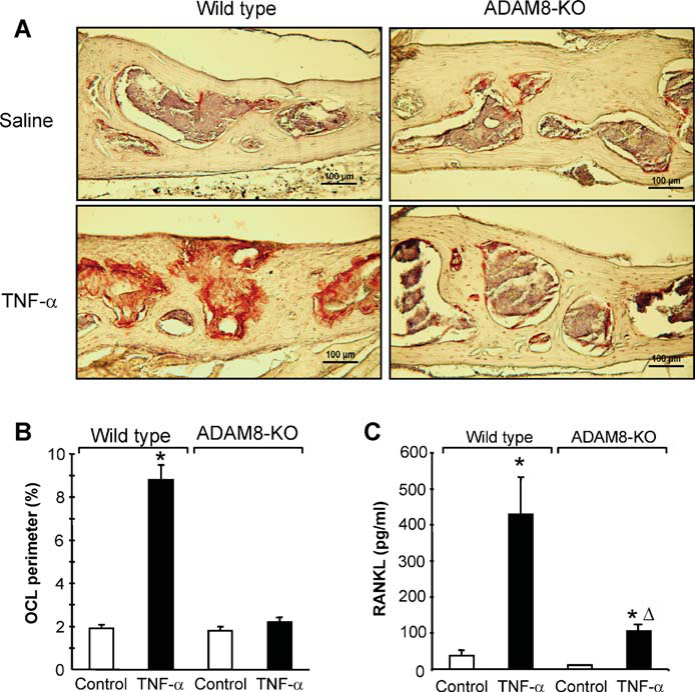
Responses to TNF-α administration in *ADAM8* KO and WT mice. Photomicrographs taken from sections of calveria from WT and *ADAM8* KO mice treated with saline or TNF-α and stained with TRAP and hematoxylin. WT mice demonstrated increased osteclastogenesis, as demonstrated by the increased numbers of TRAP^+^ (*red*) osteoclasts present in deep resorption cavities and inflammation. These responses were blunted in *ADAM8* KO mice treated with TNF-α (*p* < .01). (*B*) OCL perimeter measured in WT and *ADAM*8 KO mice treated with TNF-α or saline. TNF-α significantly increased OCL perimeter in WT but not *ADAM8* KO mice. *Significantly different from saline treatment (*p* < .01). (*C*) RANKL production by marrow stromal cells from *ADAM8* KO and WT mice. Marrow-adherent cells were isolated and cultured for 2 days with vehicle or TNF-α (10 ng/mL). The conditioned media were harvested and RANKL levels measured with an ELISA assay from R&D Systems (Minneapolis, MN, USA). Results expressed the mean ± SD for four determinations. *Significantly different from saline-treated cultures (*p* < .01). ^Δ^Significantly different from WT cultures treated with TNF-α.

We next examined the effects of loss of ADAM8 in vivo on bone remodeling both under normal conditions and when OCL formation was stimulated with TNF-α. *ADAM8* KO mice do not display a bone phenotype either in vitro or in vivo under basal conditions, and the activity of signaling pathways associated with OCL activity was similar in A*DAM8* KO and WT littermates (data not shown). However, as shown in Fig. 7*A, B*, when *ADAM8* KO mice were treated with TNF-α for 5 days, OCL formation was minimally increased, whereas in WT littermates, OCL formation was increased fivefold. Interestingly, calvarial fibrosis induced by TNF-α in WT mice was markedly decreased in *ADAM8* KO mice. Importantly, TNF-α induced less RANKL production by marrow stromal cells from *ADAM8* KO mice than in those from WT mice (Fig. 7*C*). OPG expression levels were similar in *ADAM8* KO and WT stromal cells treated with TNF-α (data not shown). Thus the blunted OCL response to TNF-α in *ADAM8* KO mice can be explained in part by changes in RANKL/OPG expression. These results demonstrate that although loss of ADAM8 did not affect normal bone modeling, it blunted the effects of TNF-α on osteoclastogenesis and inflammation.

## Discussion

The ADAMs (*a d*isintegrin *a*nd *m*etalloprotease) are a family of cell surface proteins related to class III snake venom metalloproteases.(12) The structure of ADAM8 suggests that these proteins possess both proteolytic and adhesive functions. We have reported previously that ADAM8 is the major ADAM expressed in OCLs and that it stimulates osteoclastogenesis in vitro.(1) We further demonstrated that the disintegrin domain of ADAM8 is responsible for its effects on osteoclastogenesis and that ADAM8 binds to α_9_ β_1_ on OCL precursors to induce OCL formation. ADAM8 does not bind to other integrins on OCL precursors because ADAM8 lacks an RGD sequence in its disintegrin domain.(1) Further, α_9_ β_1_ is the only receptor for ADAM8 on OCL precursors because cells lacking α_9_ integrin do not respond to ADAM8 to induce OCL formation.(9) Recent studies have suggested an important role for ADAM8 in pathologic bone destruction associated with inflammatory conditions such as rheumatoid arthritis (RA) or aseptic loosening of hip prostheses.(2,3) However, it is unclear whether ADAM8 mediates these effects in vivo or what would be the consequences of targeting ADAM8 on normal bone remodeling. Therefore, we developed transgenic mice with ADAM8 targeted to cells of the OCL lineage using the TRAP promoter and knocked out *ADAM8* by homologous recombination. As shown in Fig. 2, OCL surface and osteoclastic bone resorption was increased markedly in TRAP-*ADAM8* mice, which resulted in osteopenia. The osteopenia did not result from decreased bone formation or inhibited osteoblast activity because osteoblast surface and bone-formation rates were normal in TRAP-*ADAM8* mice. The increased OCL formation was due in part to a modest increase in OCL precursor proliferation, as evidenced both by increased [^3^H]thymidine incorporation and Ki67 staining of early OCL precursors in the cultures (Fig. 3*F*). The increased proliferation of OCL precursors in TRAP-*ADAM8* mice occurred at the early stages of the bone marrow cultures, consistent with our previous findings that OCL precursor proliferation only occurs early in bone marrow cultures induced to form OCLs.(13) Further, OCL precursors from TRAP-*ADAM8* mice treated with RANKL formed higher numbers of OCLs than WT animals at similar concentrations of RANKL. However, these OCL precursors do not appear to be hypersensitive to RANKL because the slopes of the dose-response curves for OCL precursors from TRAP-*ADAM8* mice and WT mice were similar (Fig. 3*A*). Consistent with the increased proliferation of OCL precursors from TRAP-*ADAM8* mice, Erk1/2 activation was enhanced in TRAP-*ADAM8* OCL precursors compared with WT OCL precursors (Fig. 5*B*). Further, as shown in Fig. 4*A*, modest increases of ADAM8 expression in OCL precursors from TRAP-*ADAM8* mice (∼twofold; Fig. 1*A*) resulted in enhanced expression of both c-Fos and NFATc-1 after RANKL treatment, critical transcription factors required for OCL differentiation.(14) This increase in c-Fos and NFATc-1 expression was not due to increased RANK expression on the surface of OCL precursors because c-Fms and RANK were not increased significantly in OCL precursors from TRAP-*ADAM8* mice (Fig. 4*C*). Interestingly, TRAF6 was increased in OCL precursors approximately 1.4-fold, which may be responsible for the increased activity of multiple signaling pathways and enhanced osteoclastogenesis seen in TRAP-*ADAM8* mice.

ADAM8 also enhanced OCL precursor fusion that resulted in the formation of hypermultinucleated OCLs, which contained approximately twice as many nuclei per OCL as WT OCL precursors (Fig. 3*D*). The enhanced OCL precursor fusion most likely reflects the increased expression of DC-STAMP in OCL precursors from TRAP-*ADAM8* mice, which was enhanced further with RANKL treatment (Fig. 6*A*). DC-STAMP appears to be increased selectively in TRAP-*ADAM8* OCL precursors compared with other fusion molecules associated with increased OCL precursor fusion (eg, CD44, CD48, and ATP6v0d2), which were not increased significantly in TRAP-*ADAM8* OCL precursors (Fig. 6*B, C*). Lee and colleagues(15) reported that increased NFATc-1 through upregulation of c-Fos results in increased expression of DC-STAMP. Because ADAM8 increases expression of both these transcription factors, this may explain its capacity to enhance DC-STAMP expression. Similarly, Kim and colleagues(16) also have shown that NFATc-1 induces OCL fusion via upregulation of DC-STAMP. This is so because the DC-STAMP promoter contains both AP-1- and NFATc-1-binding sites necessary for DC-STAMP expression after RANKL stimulation of OCL formation.(17) However, it is surprising that ATP6v0d2 also was upregulated only slightly (1.7-fold) in TRAP-*ADAM8* OCL precursors because NFATc-1 also can enhance expression of this fusion molecule. Further, the enhanced p38 MAPK signaling in TRAP-*ADAM8* OCL precursors also may contribute to the hypermultinuclearity of OCLs formed in marrow cultures from TRAP-*ADAM8* mice. We showed recently that enhanced p38 MAPK signaling plays a critical role in the increased nuclear number per OCL in OCLs expressing the measles virus nucleocapsid gene.(18)

OCLs formed in marrow cultures from TRAP-*ADAM8* mice had an increased bone-resorbing capacity per OCL. This increased bone-resorbing capacity per OCL appears to be multifactorial. Hypermultinucleation enhances osteoclastic bone resorption.(19) Further, increased expression of ADAM8 in OCL precursors resulted in enhanced expression of α_v_ β_3_ as well as enhanced Src and paxillin activation. α_v_ β_3_ Integrin plays a critical role in osteoclastic bone resorption through its effects on the OCL cytoskeleton and formation of the sealing zone attachment. Cells lacking α_v_ β_3_ have an impaired bone-resorbing capacity and have poor attachment to the bone surface owing to abnormal formation of the actin ring.(20)

We have shown previously that a GST-ADAM8 disintegrin fusion protein binds α_9_ β_1_ and Pyk2, which induces paxillin phosphorylation.(21) These results demonstrate that ADAM8/α_9_ β_1_ interacts with Pyk2 to enhance signaling through paxillin. Paxillin is a target for the tyrosine kinase Src, and Src plays a critical role in osteoclastic bone resorption but not OCL formation.(8) Animals lacking Src form increased numbers of OCLs, but the animals develop osteopetrosis(22) because Src kinase plays an important role in cytoskeletal organization and OCL function. Src is also involved in the regulation of OCL cell adhesion and motility, as well as cell survival. Further, a key function of Src in OCLs is to enhance rapid assembly and disassembly of the podosomes, which are specialized attachment structures on OCLs that are required for bone resorption. Pyk2 then recruits Src to the activated integrin, in particular, α_v_ β_3_, which results in phosphorylation of Cbl. Cbl in combination with phosphatidal inositol 3 kinase and dynamin play key roles in the development of osteoclastic cell polarity, cell attachment, and motility.(23,24) ADAM8 enhances the activity of both Pky2 and Src, which results in enhanced paxillin phosphorylation. Thus increased expression of ADAM8 in cells of the OCL lineage results in enhanced osteoclastogenesis owing to increased OCL precursor proliferation and fusion and enhanced bone resorption per OCL. The increased bone resorption most likely reflects increased activation of Pyk2, Src, and paxillin, as well as increased expression of α_v_ β_3_ per cell. These results suggest that increased expression of ADAM8 in OCLs associated with inflammatory bone disease plays a key role in the bone-destructive process.

Interestingly, *ADAM8*^−/−^ mice did not display a bone phenotype, did not form significantly decreased numbers of OCLs in vitro, have decreased nuclei per OCL, or display changes in DC-STAMP, p38 MAPK, or Erk signaling. These results suggest that loss of ADAM8 during development can be compensated by other molecules, which are yet to be identified. In contrast, mice that lack α_9_ integrin, the integrin receptor for ADAM8, have an OCL phenotype.(9) This suggests that the molecules compensating for the loss of ADAM8 also may bind α_9_ β_1_ integrin. Loss of ADAM8 markedly blunted the increase in OCL formation and fibrosis seen with TNF-α treatment in vivo and RANKL expression by stromal cells in vitro (Fig. 7). These results demonstrate a previously unknown role of ADAM8 in RANKL production by TNF-α. These results further suggest that ADAM8 may be a therapeutic target for blocking inflammatory bone destruction.(1) Increased expression of ADAM8 enhances OCL formation, differentiation, and bone resorption per OCL and results in osteopenia in vivo, whereas loss of ADAM8 in vivo does not significantly affect normal bone homeostasis but blocks the OCL stimulatory effects of inflammatory cytokines such as TNF-α, which is a major cytokine implicated in inflammatory bone loss.(25)
